# Is the Elite Female Athlete’s Pelvic Floor Stronger?

**DOI:** 10.3390/jcm13030908

**Published:** 2024-02-04

**Authors:** María Barbaño Acevedo-Gómez, Elena Sonsoles Rodríguez-López, Ángel Oliva-Pascual-Vaca, Tomás Fernández-Rodríguez, Ángel Basas-García, Cristina Ojedo-Martín

**Affiliations:** 1 Department of Physiotherapy, Faculty of Health Sciences—HM Hospitals, University Camilo José Cela, 28014 Madrid, Spain; acevedogomez.maria@gmail.com (M.B.A.-G.); tfernandez@ucjc.edu (T.F.-R.); cojedo@ucjc.edu (C.O.-M.); 2 Instituto de Biomedicina de Sevilla (IBiS), Department of Physiotherapy, Universidad de Sevilla, 41013 Seville, Spain; angeloliva@us.es; 3 Department of Physiotherapy, Royal Spanish Athletics Federation, 28008 Madrid, Spain; abasas@rfea.es

**Keywords:** pelvic floor, strength, dynamometry, sportswomen, sedentary, amateurs, elite

## Abstract

**Background**: Exercise can stress the pelvic floor muscles (PFMs). This study sought to assess the strength of the PFMs according to the level of physical exercise. **Methods**: An analytical observational study was carried out using digital palpation and dynamometry measurements to assess PF strength. Healthy nulliparous women were stratified according to physical exercise (physically active and sedentary) and level of physical exercise (elite, amateur, and sedentary). **Results**: Fifty-four women were analyzed, with a mean age of 25.64 (5.33) years and a BMI of 21.41 (2.96) kg/m^2^. Differences in the passive force and strength were observed between both groups of women (*p* < 0.05), and the strength was around two times higher in physically active women (*p* < 0.05). The strength was similar between elite female athletes and sedentary women (*p* > 0.05), but statistical differences were found with amateurs (*p* < 0.05). The PFM strength (*p* = 0.019) of elite female athletes (0.34 N) was almost half that of amateurs (0.63 N) and twice as strong as that of sedentary women (0.20 N). However, these differences were not significant using digital palpation (*p* = 0.398). **Conclusions**: Women who exercise generally have greater PFM strength than women who do not exercise. Physical exercise could strengthen the PFM; however, the high intensity demanded by high-level sports does not seem to proportionally increase the strength of the PFMs.

## 1. Introduction

Regular physical activity is an important health factor for all age groups. This message is increasingly present in women’s lives and has been reflected in the increase in female participation in the sports sphere. In the last Olympic Games, almost half of the athletes were women [1]. In this context, the anatomical and physiological differences that influence sports training between women and men must be analyzed. Perhaps the greatest contrast appears in the pelvic floor muscles (PFMs). The female pelvic floor (PF) may be the only part of the body where the positive effect of physical activity has been questioned [2].

Pelvic floor dysfunction (PFD) can result in conditions like urinary incontinence (UI), anal incontinence, and pelvic organ prolapse [2]. Stress urinary incontinence (SUI) is the most frequent form of PFD [3,4]. Epidemiological research indicates that SUI impacts over 40% of women [5]. Although pregnancy and delivery have been described as the main risk factors in women for the development of UI, epidemiological studies have reported that female athletes who are nulliparous experience UI episodes as well [6]. Sport has been considered a risk factor for UI in recent decades, specifically high-level sport [7,8]. The relationship between injury and performance is complex and elite sport presents specific stress factors that can potentially increase the likelihood of injury [9]. 

UI during exercise is not uncommon and a higher prevalence has been observed among athletes engaged in high-impact sports, including running and jumping [8]. The prevalence of UI in both female and male elite athletes has been investigated, with an overall prevalence of 33% being found (45.1% in females and 14.7% in males). Whilst the prevalence of UI was 5.45 times greater in females, a prevalence of UI higher than 50% was found in swimming. Swimming is a sport that has been commonly categorized as low-impact [7], therefore urine leakage may not only be linked to impact, but also to the intensity of the sport. Increased hours of training also seem to be a common risk factor for UI [10]; however, to our knowledge, there has been little research on the experiences of high-level female athletes and the relationship with the PF. 

Studies have found that experiencing UI during elite sports may be a predictor of UI later in life [11]. Elite athletes have been identified as an understudied population in research on PFD and physical activity [2]. However, the question arises as to why not all female athletes develop UI, and why many remain continent after years of sports practice. A recent systematic review [10] reported that some athletes had stopped an activity or limited their sport because PFD had had a negative effect on their competitive performance. It is necessary to design appropriate interventions for this population.

It has been suggested that high-impact sports may lead to a higher prevalence of PFD than low-impact sports. However, [8], it appears to be high intensity physical exercise that has the greatest impact on the PF. There is evidence suggesting that losses are strongly related to an increase in the intra-abdominal pressure (IAP), and that this may lead to an increased load on the PF or conversely weakness of the PF [12]. Dornowsky et al. [13], in their study on female swimmers, stated that reduced training volume and increased training intensity lead to unwanted changes in the level of electrical activity of the PFMs. The multifactorial nature of this dysfunction makes it necessary to assess the state of the PF [14]. 

Healthcare professionals often use methods such as digital palpation, ultrasound, dynamometry, electromyography, and magnetic resonance imaging due to their minimally invasive nature [15,16,17]. Currently, the joint IUGA/ICS (the International Urogynecological Association and the International Continence Society) terminology report does not designate any method as the “gold standard” for quantifying PFM strength [18]. Vaginal palpation and dynamometry are currently used to test the strength of the pelvic floor muscles (PFM) [15,19,20,21]. Vaginal palpation using the modified Oxford Grading Scale is currently one of the most widely used methods to assess PFM contraction and relaxation [22,23], despite the subjectivity involved [20]. Dynamometry measures muscle strength and is performed through a speculum that measures the maximum force generated through PFM contractions [21,22], and appears to have strong intra-examiner [19] and inter-examiner agreement [24].

Since PFD affects between one in three and one in four women, and UI occurs at rates up to five times greater in elite female athletes compared to those who do not engage in sports [25], it is relevant to understand how physical exercise impacts the PF [2]. The claim that physically fit women have a stronger PF due to regular exercise, thus preventing the development of UI, should be further questioned. Because the strength of muscle contractions is a critical factor for optimal PF function [2,4,10,20,26,27], this research sought to assess and contrast the strength of the PF according to the level of physical exercise. 

## 2. Materials and Methods

An analytical observational study was carried out using digital palpation and dynamometry measurements during a single session to assess PFM strength. This study was approved by the Ethics Committee of University Camilo José Cela (code 18_FRIU, 22 January 2022) (Madrid, Spain).

### 2.1. Participants

The research was conducted on 54 healthy nulliparous women, classified according to physical activity (physically active and sedentary) and level of physical exercise (elite, amateur, and sedentary). The classification of physical exercise included two groups: (PA) physically active women who exercised for at least 150 min a week and (S) sedentary women who exercised for less than 150 min a week or did not exercise at all [28]. The level of physical exercise was classified into three groups: (E) elite female athletes in competitions who competed at the national and international level; (A) non-elite sportswomen or amateur athletes practicing regular exercise programs and sport activities (three times, usually <6 h/week, for 10 months a year), competing at the local or regional level [29]; and (S) sedentary women who exercised for less than 150 min a week or did not exercise at all [28].

The athletes were recruited via the Centre for High-Performance Sport (CAR) of Madrid and Sport Clubs of University Camilo Jose Cela. The sedentary women were from the university population. All of them were recruited using advertisements and informative sessions. All participants received detailed information about the study, including potential risks and benefits, and provided oral and written informed consent before commencing. All data were anonymous and confidential in line with new European data protection requirements.

All of the women included were healthy, nulliparous and between 18 and 35 years of age. The exclusion criteria were pregnant women, urgent UI, pelvic organ prolapse, active or recurrent infection of the genitourinary tract, suffering from a phobia, or the impossibility of penetration or vaginal hypertonia due to which it was not possible to introduce a vaginal speculum [19,21,22], and a self-reported history of diabetes mellitus, urogynecological or pelvic surgery, pelvic irradiation, hypermobility syndrome or mobility impairments that could affect PFM morphology, stiffness or contractility. All participants attended a single session for data collection.

### 2.2. Instrumentation and Data Collection

#### 2.2.1. Sociodemographic, Medical, and Sports Practice Data

The participants completed an online questionnaire through the Survey Monkey^®^ platform (San Mateo, CA, USA), where anthropometric information (age, weight, height, body mass index (BMI)), medical history (common diseases, urinary infection, gynecological and obstetric history), and sports practice were collected through the International Physical Activity Questionnaire (IPAQ) [30]. The evaluation of UI was performed through the International Consultation on Incontinence Questionnaire-Short Form (ICIQ-SF) [31,32].

The PFM assessment was carried out during a single session in the morning, before training or physical effort.

#### 2.2.2. Pelvic Floor Muscle Assessment: Modified Oxford Grading Scale (MOS)

Each participant, with an empty bladder, was positioned supine on the stretcher with a pillow under the head and the hips. The knees were gently flexed, supported by a roller under the knees, and the lumbar spine was in a neutral position. A bidigital examination was carried out with lubricant and the global voluntary contractibility of the PFM was assessed. Participants were instructed on how to perform a correct PF contraction and were checked by means of visual inspection and palpation for correctness. The score ranged from 0 to 5 [33,34,35]. In the case of not presenting a correct contraction, the patient was instructed to perform it correctly, without parasitic contractions or pelvic movement. 

#### 2.2.3. Pelvic Floor Muscle Assessment: Intravaginal Dynamometry

PFM strength was measured using a clinical dynamometer made from acrylonitrile butadiene styrene and polycarbonate (Pelvibex^®^, Hernani, Spain, P201130449), comprising a speculum in which an inductive displacement sensor was attached to a spring of known stiffness [23]. The speculum was made from two branches opened at an angle, each with a handle and a frontal area, which was introduced to the vaginal cavity along the natural orientation of the vagina [20]. The measurements obtained showed the actual force exerted by the woman on the PFMs in newtons (N). The intrarater reliability of the dynamometer was 0.942 and the interrater reliability was 0.937. This new vaginal dynamometer to quantify PFM strength shows good reliability and validity [22].

The speculum was for individual use, and, prior to evaluation, the speculum branches were properly disinfected (Steranius 2%) and covered with a speculum-specific sheath (two-finger gloves of opaque polyethylene for gynecological examination, Legueu^®^ type) and water-soluble gel (Sulky^®^, Kennesaw, GA, USA). Two consecutive measurements of PF contraction force were recorded using the dynamometric speculum. The rest period between the two measurements was 30 s. Two values were recorded at each measurement: an initial value of the passive force (baseline strength) after opening the device for 5 seconds and the maximum voluntary strength (contraction strength) recorded by the device over a period of 10 seconds [16,20,34]. The instruction given was “squeeze the pelvic floor muscles as hard as you can” [36]. The strength of the contraction was calculated as the maximum contraction strength minus the baseline strength. All measurements were performed by a single evaluator, a physiotherapist specializing in PF rehabilitation with more than 10 years of experience.

### 2.3. Statistical Analysis

The sample size was calculated using the G*power 3.1 software (Kiel University, Kiel, Germany). Based on previous research [37,38] in the same population, considering the partial eta squared value (η2) of 0.146 obtained by means of analysis of variance, an α error probability of 0.05 and a statistical power of 0.90 were employed for sample size calculation. Considering the possibility of a sample loss of 15%, the estimated number of subjects was 40 physically active women. The prevalence of a sedentary lifestyle in women between 18 and 35 years old is 21% [39], and so the estimated number of subjects was 14 sedentary women.

Data were analyzed using the IBM Statistics Package for Social Science, v.26 (IBM Corp, New York, NY, USA). Data were provided as the mean and standard deviation, along with 95% confidence intervals (95%CI). When appropriate, data were provided as percentages. Before performing the analysis, Shapiro–Wilk tests were used to check the normality of the variables (*p* > 0.05). The dynamometric value was computed for each group and compared using the Kruskal–Wallis test for analysis of the level of physical exercise, and the U Mann–Whitney test was used to compare physically active and sedentary women. The Games–Howell post hoc procedure was used to compare differences between groups regarding the level of physical exercise. In all tests, the effect size (η^2^) was calculated. The general linear model procedure generated an effect size, known as partial **η^2^**, categorized as small (0.01), medium (0.06), and large (0.14) [40,41]. Bivariate correlations among quantitative variables were assessed through Pearson’s coefficient. The level of confidence was set at 95% and significance was set at *p* <  0.05.

## 3. Results

Sixty nulliparous females were recruited, and six of them were excluded from the analysis due to vaginismus and an inability to insert the dynamometric speculum; one was an elite athlete, another was an amateur athlete, and four women were from the sedentary group. The final sample comprised 54 nulliparous females with a mean age of 25.64 (5.33) years and a BMI of 21.41 (2.96) kg/m^2^. None were overweight. Five women presented with constipation, fifteen experienced UI, and none had undergone gynecological surgery. Only one Black woman participated in this study. Thirteen female athletes from different sports were analyzed (badminton (*n* = 2), gymnastics (*n* = 3), and athletics (*n* = 8)), who dedicated more than 3 h a day to training. Twenty-five sportswomen from athletics (*n* = 18) and rugby (*n* = 7) formed the amateur group. 

According to the level of physical activity (Table 1), physically active (*n* = 38) and sedentary (*n* = 16) women were analyzed. The differences between the initial value of the passive force (baseline strength) and the strength were observed between both groups of women (*p* < 0.05), and the strength was around two times higher among the physically active women (*p* < 0.05).

**Figure 1:**
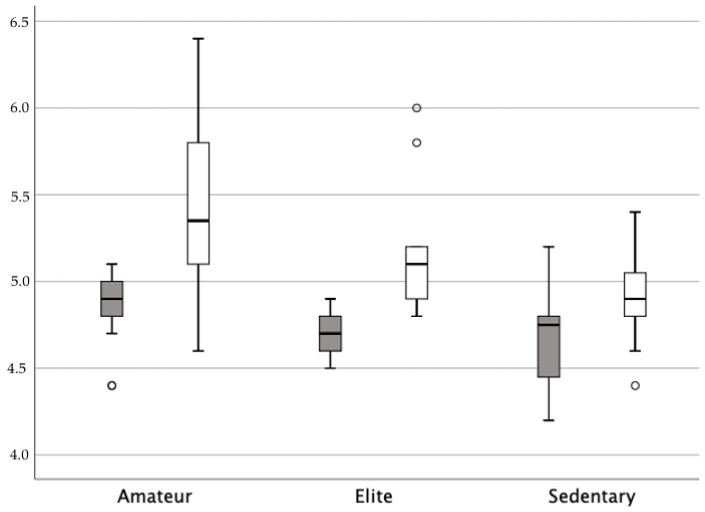
Representation of PFM strength acording to level of physical exercise. The graph represents the passive force (dark grey) and the maximum voluntary strength (white) in Newtons.

According to the level of physical exercise, Table 2 shows the PFM strength measured with the dynamometric speculum and the score on the modified Oxford Grading Scale for each group. Age and BMI were similar in the three groups. The analysis was significant (*p* < 0.05), indicating differences in baseline and contraction strength measurement with the dynamometric speculum. Similar values were obtained in the first and second measurement in all groups (*p* > 0.05). In the post hoc analysis, the passive force was higher in amateurs (*p* = 0.008). The strength and maximum voluntary strength were not significant different between elite female athletes and sedentary women, but statistical differences were found between amateur sportswomen and elite athletes and sedentary women. The highest strength measurement was 0.63 N among the amateur sportswomen, and the lowest value was 0.20 N among the sedentary women, followed by 0.34 N among the elite female athletes (Figure 1). However, the analysis showed no significant difference in the score on the MOS (*p* = 0.398). Significant direct associations were found between the maximum voluntary strength and the strength between measurements and the score on the MOS (*p* < 0.05).

Three sedentary women and four elite athletes and eight amateurs reported stress urinary incontinence through ICIQ-SF. There were no differences between women with or without urinary leakage regarding their baseline and contraction strength measurements (*p* > 0.05).

## 4. Discussion

The aim of this study was to assess the strength of the PF according to the level of physical exercise. The results suggest that physical exercise seems to modify the passive component of the PFM, and strength was around two times higher among the physically active women versus the sedentary women. According to the level of physical exercise, small differences were observed in passive forces. Strength was greater in amateur sportswomen than in elite athletes, and above the average for sedentary women. Therefore, it seems that the practice of physical exercise is beneficial for the PFM in terms of strength, but this may be conditional in high-level competition.

Regarding the evaluation used, there were no significant differences between the first and second measurements, which could be interpreted as indicating that there was sufficient rest time in between them. Our protocol used a 30 s rest period between the two measurements. Similar results were obtained in prior studies using 30-s, [19] one-minute [37] two-minute [42,43], and three-minute [24] rest periods [42]. The moderate to high repeatability of the measurements allowed the dynamometers to be considered a reliable tool for assessing the function of the PFM [44].

Our study population comprised nulliparous females with an average of age of 25.64 (5.33) years. This represented a difference from previous studies [45], which did not consider a younger population with these particularities. Our results were corroborated by Araujo et al. [46], who compared PFM strength among 49 female athletes versus 44 sedentary women and showed that the athletes had a higher capacity to contract the PFMs. Although their population did not cover elite athletes, their findings corroborated the results of the present study, which also found a significantly greater capacity to contract the PFMs among athletes than sedentary women, as assessed by a manometer.

Regarding our findings, two main conclusions are worth highlighting. The first was that the amateur population seems to have a greater PFM strength than the elite and sedentary population. To our knowledge, no publications have compared high-level athletes with other populations. Two opposing hypotheses may explain our results. A recent review [2] noted that exercise may either strengthen or overload and stretch the PFMs. Women who exercise generally have similar or stronger PFM strength and a larger cross-sectional area of the levator muscles than women who do not exercise. However, exercise intensity is also an important factor in UI, as UI is linked to the high intensity demanded by high-level sports [8,47]. In our study, we did not find significant differences between contraction strength and urinary symptoms, perhaps because the sample size was small. On the other hand, this same review stated that mild to moderate physical activity, such as walking, decreases the risk of UI, in congruence with our results with respect to the PFM strength in amateur women.

There were significant differences demonstrating a strong clinical effect in the measurement of passive and active elements, as well as in muscular strength, between the three groups. PFM tone is defined as the resistance provided by an innervated muscle when stretching is applied. Muscle tone is composed of an active (electrogenic) component and a passive (viscoelastic) component [48]. We assessed the passive component as the baseline strength in the three groups, and the contribution of the passive component to PFM tone in amateurs was higher. Morin et al. [49] found similar results for passive PFM tone (4.61 ± 1.72) in the control group compared to a group of women with vestibulodynia (3.75 ± 1.48), but they did not provide data on physical activity. The impact of task familiarization on passive PFM force generation is also unknown and may be relevant [49]. It potentially explains why the second measurement of passive force, although it continued to be greater in amateurs, was not significant between groups. However, Czyrnyj et al. [50] did not find any effect of task familiarization in any active or passive PFM properties measured by intravaginal dynamometry in nulliparous women. We also did not find a difference in the second measurement. 

The active component of PFM tone was assessed according to contraction strength. The PFM strength of elite female athletes (0.34 N) was almost half that of amateurs (0.63 N) and twice as strong as that of sedentary women (0.20 N), with similar results for the second measurement. The differences between the groups according to the physical activity performed differed in intensity from previous studies. Araujo et al. [46] found the capacity of PFM contraction to be 51% higher in athletes compared with sedentary women, as assessed by perineometer. Dos Santos et al. [51] found that incontinent athletes had 25% greater PFM strength, as assessed by a perineometer, than continent athletes, suggesting that UI in athletes is not due to PFM weakness. Arbieto et al. [12] found that the capacity to contract the PFMs was 21.3% higher among athletes than among non-athletes, as assessed by a manometer. 

The results of the MOS showed no significant differences. The Oxford Grading Scale is a subjective method with low test–retest and inter-rater reliability [52] and seems to be a poor assessment method to draw conclusions on the PFM. Therefore, healthcare professionals should use a combination of tools to optimize the diagnosis of the PFM.

The main study limitations could be primarily attributed to the small and heterogenous sample size, which may have affected the generalizability of the results. However, the sample size was calculated and based on previous research [37,38] in the same population. It was difficult for us to find sedentary participants, since the population was nulliparous university women where sports practice is widespread. Larger study groups could provide more representative data. Another limitation in the PFM assessment was that the accessory muscle contraction was monitored via visual inspection. It may be better to monitor the co-contracting muscles with other evaluation methods (electromyography or ultrasound). Regarding the assessment position, all measurements were performed in a supine position instead of a standing position, in which the PFM is more linked to activities of daily living and sports routines in our population. Our dynamometer was opened at an angle, which caused a higher opening pressure applied to deeper vaginal tissues. This can cause discomfort and thereby limit the measurement of PFM force at different muscle lengths [42]. In our study, women for whom inserting a vaginal speculum was not possible or who felt discomfort were excluded.

Future research could extend these findings to larger sample sizes with pelvic floor dysfunction and could quantify the intensity of physical exercise. It would also be interesting to monitor the muscles working together with the PFMs. The elite women athletes in our sample participated in competitions such as the Olympic Games, and it is necessary that science continues researching urogynecological factors for women in sports. We certainly feel that the PFM deserves more attention so that athletes can be made more aware of it and its relevance to their physical demands.

## 5. Conclusions

Women who exercise generally have higher PFM strength than women who do not exercise. Physical exercise could strengthen the PFM; however, the high intensity demanded by high-level sports does not seem to proportionally increase the strength of the PFM. The PFM strength of elite female athletes was almost half that of amateurs and twice as strong as that of sedentary women. Therefore, exercise intensity also appears to be an important factor for the PFMs. Sports and healthcare professionals need to pay attention to training and strengthening sportswomen’s PFMs to adapt them to their physical demands and reduce the occurrence of pelvic floor dysfunctions.

## Figures and Tables

**Table 1:** Pelvic floor muscle strength measured with the modified Oxford Grading Scale and dynamometric speculum, according to level of physical exercise.

	Physically Active (*n* = 38)		Sedentary (*n* = 16)		Between Groups *p* Value *
	Mean	SD	Mean	SD	
Strength (Modified Oxford Grading Scale)	3.68	0.57	3.43	0.51	0.175
Baseline strength (N) 1st measurement	4.79	0.11	4.63	0.26	<0.001
Baseline strength (N) 2nd measurement	4.82	0.18	4.68	0.29	0.048
Contraction strength (N) 1st measurement	5.32	0.56	4.83	0.21	<0.001
Contraction strength (N) 2nd measurement	5.38	0.50	4.89	0.25	<0.001
Strength (N) 1st measurement	0.53	0.55	0.20	0.14	0.047
Strength (N) 2nd measurement	0.56	0.50	0.21	0.15	0.021

N, Newtons. Data from continuous variables are presented as the mean (standard deviation). Significance level was set at *p* < 0.05. (*) Difference between groups according to the *p* value based on the U Mann–Whitney test.

**Table 2:** PFM strength measured with the modified Oxford Grading Scale and dynamometric speculum, according to the level of physical exercise.

	Physically Active (*n* = 38)				Sedentary (*n* = 16)		Between Groups *p* Value *	η^2^
	Amateur (*n* = 25)		Elite (*n* = 13)					
	Mean (SD)	95% CI	Mean (SD)	95% CI	Mean (SD)	95% CI		
Age (years)	26.08 (5.31)	(23.89–28.27)	25.08 (5.72)	(21.62–28.53)	25.19 (5.23)	(22.40–27.97)	0.865	-----
BMI (kg/m^2^)	20.93 (2.44)	(19.92–21.94)	20.12 (1.36)	(19.3–20.95)	23.03 (3.91)	(20.95–25.12)	0.076	-----
Strength (Modified Oxford Grading Scale)	3.68 (0.55)	(3.45–3.9)	3.69 (0.63)	(3.11–4.07)	3.43 (0.51)	(3.16–3.71)	0.398	-----
Baseline strength (N) 1st measurement	4.83 (0.09)	(4.79–4.87)	4.70 (0.10)	(4.63–4.77)	4.63 (0.26)	(4.49–4.76)	0.001	0.240
Baseline strength (N) 2nd measurement	4.87 (0.18)	(4.8–4.95)	4.72 (0.12)	(4.64–4.79)	4.68 (0.29)	(4.52–4.83)	0.004	0.158
Contraction strength (N) 1st measurement	5.45 (0.61)	(5.20–5.70)	5.05 (0.28)	(4.88–5.22)	4.83 (0.21)	(4.72–4.95)	<0.001	0.260
Contraction strength (N) 2nd measurement	5.50 (0.52)	(5.29–5.72)	5.14 (0.35)	(4.92–5.36)	4.89 (0.25)	(4.75–5.02)	<0.001	0.290
Strength (N) 1st measurement	0.62 (0.63)	(0.35–0.88)	0.34 (0.28)	(0.17–0.51)	0.20 (0.14)	(0.12–0.28)	0.039	0.142
Strength (N) 2nd measurement	0.63 (0.55)	(0.40–0.86)	0.42 (0.33)	(0.22–0.62)	0.21 (0.15)	(0.13–0.29)	0.019	0.147

N. Newtons. Data from continuous variables are presented as the mean (standard deviation). Significance level was set at *p* < 0.05. (*) Difference between groups according to the *p* value based on the Kruskal–Wallis test.

## Data Availability

The data presented in this study are available upon request from the corresponding author. The data are not publicly available due to privacy and ethical restrictions.
